# Educational Mobility and Attitudes Towards Migration from an International Comparative Perspective

**DOI:** 10.1007/s12134-022-00977-8

**Published:** 2022-08-08

**Authors:** Mathew J. Creighton, Daniel Capistrano, Monika da Silva Pedroso

**Affiliations:** 1 Dublin School of Sociology, University College, Dublin, Ireland; 2 UCD Geary Institute for Public Policy, Dublin, Ireland; 3 University College Dublin School of Education, Dublin, Ireland

**Keywords:** Educational stratification, Mobility, European social survey, Public opinion, Opposition to immigration

## Abstract

This work considers the role of intergenerational educational mobility in shaping attitudes towards immigration. Two substantive questions drive this work. First, does the experience of stagnant or downward educational mobility result in negative attitudes towards immigration? Second, are perceptions of immigration shaped by the relative importance of parental (i.e. origin) and one’s own (i.e. destination) level of education? We deploy six waves of the European Social Survey (ESS) to assess how upward, downward and stagnant intergenerational educational mobility shape attitudes towards immigration across 31 countries. Results show that upward educational mobility can moderate antipathy towards immigration, but this is more applicable in country-contexts where parental education is less relevant. In other words, education matters for our understanding of how immigration is viewed, but its role must be framed in a way that takes into account multiple generations.

Immigration is at the fore of contemporary social and political debates. In particular, the determinants and implications of antipathy towards migrants and immigration has garnered extensive interest in the literature (Ceobanu & Escandell, 2010; Hainmueller & Hopkins, 2014) One proposed determinant of the observed recent increase in anti-immigrant sentiment focuses on socioeconomic mobility—or the lack thereof (Jackson & Grusky, 2018; Paskov et al., 2021). This perspective highlights the frustration among those who experienced limited or stagnant intergenerational upward mobility—occupational or educational. In this work, we bridge standard explanations of anti-immigrant sentiment, which are shaped by individual-level concerns over economic and sociocultural threat, with models that account for measures of intergenerational mobility. Material (e.g. employment, wages, attained education and wages) or sociocultural (e.g. religiosity, political orientation and nationalism) considerations are not ignored, but compliment a model in which one’s attitudes towards migrants are patterned by educational origins and destinations—within and across country contexts.

We use six rounds of the European Social Survey (ESS) that allow us to consider a substantial time series to capture how upward, downward and stagnant intergenerational mobility shapes attitudes towards immigration in 31 countries. To model mobility as predictor of attitudes towards migrants, we implement the diagonal mobility model (DMM) or diagonal reference model (DRM). This perspective offers a number of theoretical (Blau, 1956; Blau & Duncan, 1967; Sobel, 1981) and methodological advantages (Hendrickx et al., 1993; Schuck & Steiber, 2018; Sobel, 1981, 1985).^1^ The key innovation of the model is the ability to delineate the role of mobility from that of the origin and destination statuses (Van der Waal et al., 2017).

Our approach is driven by two substantive questions. *First*, does the experience of stagnant or downward mobility result in negative attitudes towards immigration relative to those who experience upward intergenerational mobility? *Second*, to what extent does the relative influence of origin or destination education on attitudes towards immigration vary by country context? To foreshadow our findings, we show that mobility patterns variation in attitudes towards immigration even after accounting for a number of socioeconomic and demographic concerns. Moreover, we find that country contexts where one’s own education is relatively influential—relative to parents’ education—are defined by a greater shift towards a more tolerant perspective on immigration when comparing the upwardly mobile to other mobility patterns. We also urge caution when interpreting the findings by underlining some important caveat, which offer pathways for future research.

## Education, Mobility and Attitudes Towards Migrants

Work on attitudes towards out-groups (e.g. migrants, ethnic/religious minorities) consistently finds a positive association between education and general tolerance (Capistrano, 2020; Creighton et al., 2019; Jenssen & Engesbak, 1994; Knudsen, 1995; Kuppens & Spears, 2014; Tolsma et al., 2009; Velásquez & Eger, 2022), liberal values (Bobo & Licari, 1989; Hello et al., 2004).^2^ Rather than the content of an educational experience, less is often associated with social and cultural capital derived from the broader experience that educational institutions support and/or the selection into education by those who come from more open social origins (Weil, 1985). In addition, evidence suggests that the extent to which education moderates anti-immigrant sentiment varies by context, which are defined by educational/institutional environment and prevailing social norms (Kunovich, 2004; Tolsma et al., 2009).

Individual-level determinants of anti-immigrant sentiment are widely studied (for an extensive review of the literature, see Ceobanu and Escandell (2010) and Hainmueller and Hopkins (2014)). The literature often focuses on threat—material and/or sociocultural, perceived or experienced—by non-immigrant members of a destination context (Gorodzeisky & Semyonov, 2020; Stawarz and Müller, 2020; Creighton et al., 2015; Creighton and Jamal, 2021; Creighton et al., 2022). The measure of interest is often education, generally interpreted as human capital (materialist perspective) or cultural/social capital (sociocultural perspective). Material threat is theorised as a reaction to real/perceived competition for employment or wages (Borjas, 1999; Gorodzeisky and Semyonov, 2020; Hainmueller and Hiscox, 2010; Mayda, 2006; McLaren & Johnson, 2007; Scheve & Slaughter, 2001). Sociocultural threat is theorised as identity-based, characterized by perceived incompatibilities based on religion, norms, race, ethnicity, gender and language—among others (Hainmueller & Hiscox, 2007; Manevska & Achterberg, 2013; Sniderman & Hagendoorn, 2007; Sniderman et al., 2004; Strabac & Listhaug, 2008). To be clear, these perspectives are not mutually exclusive (Hello et al., 2002; McLaren, 2003; Rustenbach, 2010).

Recent work has examined whether education can be seen as a determinant of attitudes towards immigration. Research in Germany concludes that lower levels of education translates in a amplification of the perception of ethnic conflict, particular after certain events in the life course (Kratz, 2021). Other work, also in Germany, finds that education is directly linked to greater tolerance of immigration (Margaryan et al., 2019). In contrast, work in Sweden concluded that education has no direct causal relationship with attitudes towards immigration (Finseraas et al., 2018). Other work in Ireland (Creighton et al., 2022) and the Netherlands points out that the more educated are also more likely to conceal intolerance in direct interactions (e.g. surveys). Some suggest that superficial interaction in school can have mixed results in terms of moderating anti-immigrant sentiment (Bentsen, 2022). What is less understood is the role of educational mobility—as opposed to attained education, which is the marker most often taken into account at the individual level. Although absent for standard models of anti-immigrant sentiment, educational mobility is a core concern of social stratification research more broadly and its role in a variety of attitudinal outcomes has been explored (for an extensive overview see Hendrickx et al., 1993).

Starting with efforts to understand the reproduction of status (Blalock, 1967; Goldthorpe et al., 1987; Sorokin, 1927), interest in the wider implications of mobility has considered links with emotion (Friedman, 2016), well-being/mental health (Chan, 2018; Houle & Martin, 2011; Nikolaev & Burns, 2014; Schuck & Steiber, 2018; Zang & de Graaf, 2016), consumption (Paulson, 2018), politics (Breen, 2001; Dalhouse & Frideres, 1996; Weakliem, 1992) and core demographic behaviours (Hope, 1971; Sobel, 1985). In addition, research across the social sciences, has considered the meaning of intergenerational mobility for social and economic stratification (Becker et al., 2018; Torche, 2015). Some work has also considered intergroup relations, although not immigration specifically, finding that upward mobility can moderate antipathy in the Netherlands (Tolsma et al., 2009). The general pattern is that upward mobility has positive implications, but the relationship is sometimes tenuous relative to theoretical expectations.

## Education, Attitude Formation and Socialisation

When considering intergenerational educational mobility, it is crucial to understand that the relative importance of parents’ and one’s own education varies by context and moment in the life-course (Borgonovi & Pokropek, 2019). During early childhood and adolescence, when many are co-residing with their parents, the role of parental education and the constellation of norms associated with it are relatively more prominent (Darling & Steinberg, 1993; Miller & Glass, 1989; Moen et al., 1997). Some have shown that interaction with previous generations, proxied by physical proximity, is the key to understand why parental (and grandparental) education matter for children (Torche, 2015; Zeng & Xie, 2014). That does not mean that educational origins, which are the foundation for a variety of “capitals” (e.g. cultural; Bourdieu [1986]; Scherger & Savage [2010]), fade away as one ages. Instead, evidence suggests that the shadow of social origins extends well into adulthood, continuing to shape children’s social, economic, political (Malloy et al., 2021) and educational progression (Erola et al., 2016; Hess & Torney, 1967). In fact, these norms can solidify with attitude stability increasing with age (Alwin & Krosnick, 1991; Min et al., 2012). Of note, the extent to which parental influence transmits to a new generation varies notably by the educational background of the parents (Van der Slik et al., 2002) and gender (Beller, 2009; Cordero-Coma & Esping-Andersen, 2018). Despite this variation, many underline how social origins, particularly education, remain the most important determinants of the mobility of the next generation (Björklund & Salvanes, 2011; Hauser & Featherman, 1976; Sieben et al., 2001). In other words, the way that parents project themselves in the world and simultaneously on their children significantly influences descendants’ learned repertoire of attitudes and behaviours—in the short and long term.

One’s own education emerges as the educational process unfolds, but acts in greater independence from parental influence as one approaches relative independence. As with parental education, respondent’s own education conditions socio-cultural attitudes, norms, values and behaviours. Attitudinal change is evident later in life, although considerable additional inputs are expected given the role of parents as the central agent of socialisation in childhood, particularly if emergent perspectives conflict with parental perspectives (Dalhouse & Frideres, 1996; Miklikowska, 2017). Greater levels of schooling can lead to an increase in the acceptance of more tolerant values and norms—at least in societies where tolerance is the predominant social norm and is reflected in educational settings (Tolsma et al., 2009). In other words, education can enhance the development of open mindedness and cognitive competence and mitigates authoritarian attitudes, subsequently collaborating to the fostering of a more tolerant orientation towards ethnic and racial out-groups (Hello et al., 2006; Tolsma et al., 2009). In terms of material considerations, evidence consistently shows that educational attainment improves labour market position and moderates the perception of ethnic competition and feelings of threat (Hello et al., 2006; Tolsma et al., 2009).

This work seeks to understand the role of educational mobility—upward and downward—in shaping sentiment towards immigration. Moreover, we consider the context-specific relative importance of origin/parent’s and destination/respondent’s education. This permits an assessment of the extent to which parental education, defining influences earlier in the life-course, is (or is not) relatively more determinant of attitude formation than one’s own education, which has been pointed out by some (e.g. Fosse & Pfeffer, 2019).

### Hypotheses

Drawing from efforts to understand the role of education in shaping attitudes towards immigration and the broader literature on social mobility, we consider a number of theoretical expectations. First, downward and stagnant intergenerational educational mobility define life-course experiences where the social and economic outlook of the future is less optimistic or certain. Immigration, intimately linked to contemporary patterns of global social and economic interconnectedness, is perceived to reflect an advantage from which some have been excluded. The result is an embrace, rooted in material concerns—real or perceived—of a more restrictive posture towards immigration, which is reflected in the following expectation: *Individuals who have attained the same or less education than their parents are expected to be less supportive of immigration that those who have experienced upward educational mobility* (H1).

As noted, parental inputs are more influential early in the life course. Given the large literature finding that more education results in a relative more tolerant perspective on immigration, we suggest the following expected relationship based on distinct mobility patterns and, moreover, the relative importance/weight of parental education: *Educational mobility moderates attitudes towards immigration less, reflected in a smaller gap between those who do and do not experience upward mobility, in contexts where parental education is relatively more influential* (H2a). As higher levels of schooling occur later in the life-course, greater upward mobility can shift the importance away from social origins (i.e. parental education) towards one’s own experience: *Educational mobility matters more, reflected in a greater gap between those who do and do not experience upward mobility, in moderating attitudes towards migrants in contexts where parental education is relatively less influential* (H2b).

## Methods

### Data Source/Sample

This work benefits from six rounds of the European Social Survey (ESS 2018i; 2016; 2014; 2012; 2010; 2008). Encompassing 31 national samples, the overall goal of the ESS is the measurement of attitudes, beliefs and behavioural patterns in a way that are comparable across countries and across rounds. First collected in 2001 and awarded a European Research Infrastructure Consortium (ERIC) in 2013, the ESS is fielded every 2 years by participating countries. The rounds used in this work (described in greater detail below) include all data collected since 2008 (ESS 2019; 2018a; 2018b; 2018c; 2018d; 2018e) and are available free-of- charge to all registered^3^ users.

All the 31 countries that took part in the survey were included in this research. Countries took part in the ESS on a voluntary basis, with 15 of them participating in all the six rounds analysed and 2 of them taking part in only one round. Although the ESS participating countries represent the majority of countries in Europe, the scope of this study and its conclusions are limited to those countries sampled in the ESS.

The key outcome of interest is respondent’s attitudes towards immigration, their completed education and their parents’ education. Although the ESS has been collected every 2 years since 2002—a total of nine rounds—we limit our sample to the 4th, 5th, 6th, 7th, 8th and 9th rounds. These last six rounds are the only rounds that included harmonised education measures for all respondents and their parents, which is a minimal requirement to model intergenerational educational mobility (ESS, 2009), resulting in a baseline sample of 287,701 respondents. This sample was further restricted to respondents aged 26 years or more to capture the point in the life-course when further educational attainment is unlikely, which results in a sample of 242,054 respondents. To account for known variation in in-group and out-group perceptions of immigration (e.g. Bazo-Vienrich & Creighton, 2017), we limit the sample to respondents who were citizens of the country in which they were interviewed, resulting in a sample of 233,216 respondents.

#### Missing Data

A further 10,633 respondents without information on attitudes towards immigration, which is the outcome of interest, and 25,221 respondents missing socioeconomic data (education and employment) or demographic data (sex, age, migrant status, urbanicity) were excluded from the analysis. None of these exclusions by variable account for more than 1% of the total sample, except for parental education (Table 6). The listwise deletion was preferred in this case due to the large sample size and considering that multiple imputation does not guarantee to improve accuracy and precision of the estimators given the specification of our model (Arel-Bundock & Pelc, 2018). The final analytic sample includes 197,362 respondents (for a detailed description, see Appendix 1).

### Explanatory Variable: Educational Attainment

Education is a notoriously difficult measure for international comparative research (Schröder & Ganzeboom, 2014). Europe includes a large number of distinct educational contexts and assessing mobility within each requires the creation of broader categories of educational attainment to capture comparable mobility patterns. Starting with the 4th round,^4^the ESS uses country-specific experts to generate the seven,^5^ consistently defined categories for each country where the survey is conducted (ESS 2019; Appendix 1). In the case of mobility, the initial seven categories can be further reduced to three broad, but substantively meaningful transitions—low, medium and high. Low includes those who completed lower secondary or less, which includes those that did not attend school and primary-school leavers (i.e. ISCED I, ISCED II). Medium consists of those with either some form of secondary school or vocational training beyond lower secondary (i.e. ISCED IIIb, ISCED IIIa, ISCED IV). High includes any form of tertiary schooling (i.e. ISCED V1, ISCED V2).

In addition to the respondents’ highest attained education, the ESS indirectly records education for one or both parents, which is consistently coded to be comparable across contexts and rounds. To best capture the origin educational context with a single measure, we elected to use the highest education of both parents or the education of a single parent in cases where relevant. In the end, each respondents’ educational mobility is shaped by two, three-category measure of attained education. These two measures define an educational origin (i.e. parental education) and destination (i.e. respondent’s education).

### Outcome Variable: Attitudes Towards Immigration

Attitudes towards migrants encapsulate a number of distinct dimensions ranging from the economic/material to the social/cultural. The European Social Survey provides three related measures to assess the economic, cultural and general perception of migrants.^6^ For each dimension, respondents are offered an 11-point scale from which to express their preferences with responses ranging from (Q1) *bad for the economy*/(Q2) *cultural life undermined*/(Q3) *worse place to live* to (Q1) *good for the economy*/(Q2) *cultural life enriched*/(Q3) *better place to live*. For the sake of parsimony and clarity, we have selected the last question (Q3) that assesses the general perception of migrants. This question is also highly correlated with both the economic (*r*(186,303) = 0.67, *p* < 0.001) and the cultural perceptions (*r*(186,912) = 0.72, *p* < 0.001). In the original wording of the question, higher values indicated more positive views of immigration. We considered a model of opposition to immigration to be more easily interpreted if the scale were reversed and all responses were re-coded such that 11 was the most negative and 0 the most positive.

The resulting measure is interpretable as higher values indicating a respondent considers immigration to be worse for the general wellbeing of the country in which they reside. Table 1 reports the average perception of immigration by mobility combination (e.g. Low education to low education). Even with the basic descriptive statistics, a general pattern emerges such that negative sentiment declines as parental (i.e., origin) and one’s own (i.e. destination) education increase. Specifically, across all countries the average response among children of the least educated who share the same level of schooling is 5.68. This decreases to 5.35 for children of the most educated who attain similar levels of schooling.

**Table 1:** Descriptive statistics by country—all ESS rounds by countries

Country	Low-low Mean (SD)	Med.-Med	High-High	Overall	Observations
Austria	6.14 (2.20)	5.45 (2.10)	4.38 (2.20)	5.78 (2.30)	4954
Belgium	5.69 (2.20)	5.39 (2.00)	4.43 (1.80)	5.18 (2.10)	7640
Bulgaria	5.28 (2.70)	5.21 (2.50)	4.96 (2.20)	5.14 (2.50)	5039
Switzerland	5.01 (1.80)	4.32 (1.70)	3.80 (1.90)	4.61 (1.90)	6190
Cyprus	6.58 (2.40)	6.20 (2.60)	5.51 (2.20)	6.39 (2.40)	2343
Czechia	6.28 (2.30)	5.92 (2.10)	5.77 (2.10)	6.07 (2.20)	9834
Germany	5.39 (2.20)	4.45 (2.20)	3.66 (2.00)	4.77 (2.20)	12,853
Denmark	4.99 (2.20)	3.54 (2.00)	3.02 (1.70)	4.17 (2.20)	4846
Estonia	6.43 (2.10)	5.59 (2.20)	4.79 (1.90)	5.57 (2.10)	6918
Spain	5.38 (2.20)	4.56 (2.10)	3.90 (2.10)	4.99 (2.20)	7646
Finland	5.05 (2.10)	4.70 (2.00)	3.57 (1.90)	4.46 (2.00)	8177
France	6.00 (2.20)	4.73 (1.90)	4.25 (2.00)	5.30 (2.20)	8784
UK	5.92 (2.40)	4.87 (2.30)	3.23 (2.10)	5.03 (2.50)	7581
Greece	7.75 (1.90)	6.87 (1.90)	6.27 (2.40)	7.40 (2.10)	2095
Croatia	5.49 (2.40)	4.94 (2.20)	4.36 (2.10)	5.18 (2.40)	2237
Hungary	6.39 (2.20)	5.70 (2.10)	5.20 (2.00)	6.05 (2.10)	6321
Ireland	5.32 (2.50)	4.15 (2.20)	2.89 (2.00)	4.51 (2.50)	8798
Israel	5.53 (2.60)	4.91 (2.40)	4.27 (2.50)	4.97 (2.60)	6290
Iceland	3.65 (2.00)	3.33 (1.80)	2.69 (1.80)	3.16 (1.90)	1250
Italy	6.85 (2.40)	5.88 (2.40)	4.94 (2.40)	6.28 (2.40)	4655
Lithuania	5.45 (2.20)	5.26 (2.10)	4.65 (1.90)	5.20 (2.10)	5503
Netherlands	4.94 (1.90)	4.31 (1.60)	4.03 (1.60)	4.64 (1.80)	8378
Norway	5.06 (2.00)	4.73 (2.00)	3.76 (1.80)	4.48 (2.00)	6970
Poland	4.54 (2.10)	4.15 (2.00)	3.70 (2.00)	4.28 (2.10)	7097
Portugal	6.11 (2.00)	5.28 (2.20)	4.33 (2.20)	5.84 (2.10)	7194
Serbia	5.79 (3.20)	5.16 (2.90)	3.96 (2.60)	5.60 (3.10)	1593
Russia	6.73 (2.50)	6.65 (2.30)	6.41 (2.20)	6.64 (2.30)	6887
Sweden	4.42 (2.10)	3.48 (2.20)	2.42 (1.90)	3.55 (2.20)	5119
Slovenia	6.20 (2.30)	5.27 (2.10)	4.14 (2.10)	5.56 (2.30)	6035
Slovakia	5.91 (2.00)	5.67 (1.90)	5.75 (2.10)	5.73 (2.00)	4239
Ukraine	6.24 (2.50)	5.59 (2.40)	5.45 (2.40)	5.72 (2.50)	3978
Mean (SD)/Total	5.68 (2.30)	5.66 (2.40)	4.63 (2.40)	5.35 (2.40)	197,362

Source: European Social Survey (ESS 2018i; 2016; 2014; 2012; 2010; 2008; 2006; 2004; 2002)

The mean response reported above is derived from weighted data (*dweight*). Please see Appendix 1 for complete details about the sample selection. A replication package is available at [https://bit.ly/3ecrv4I]

### Explanatory variable

The primary explanatory variable is educational mobility. Depending on one’s origin and destination, upward, stable and downward educational mobility patterns are possible, which are disaggregated by sociodemographic attributes of the respondent in Table 2. Of women, 36.2% are upwardly mobile, 56% reproduce the attained education of their most educated parent and 7.8% move downward in terms of education. Men experience a substantively identical pattern.

**Table 2:** Descriptive statistics of variables in multivariate model—all ESS rounds/years and countries

	Opposition to immigration (0) Less…..(10) More		Educational mobility Downward Stable Upward			Observations
	Mean (SD)	%	Row %			
Sex						
Female	5.42 (2.40)	53.3	07.8	56.0	36.2	105,225
Male	5.27 (2.40)	46.7	08.6	56.3	35.1	92,137
Age						
[26–44]	5.19 (2.40)	34.7	11.7	51.4	36.9	68,403
[45–64]	5.34 (2.40)	41.6	06.9	54.4	38.8	82,099
65 +	5.60 (2.40)	23.7	05.4	66.1	28.5	46,860
Migrant status						
Native	5.42 (2.40)	86.9	07.9	56.4	35.7	171,449
1st generation	4.71 (2.50)	06.1	10.3	55.7	34.1	11,955
2nd generation	5.00 (2.40)	07.1	09.8	52.9	37.3	13,957
Urbanicity						
Urban	5.32 (2.40)	63.8	09.0	53.8	37.2	125,977
Rural	5.40 (2.30)	36.2	06.9	60.1	33.1	71,385
Employment						
Employed	5.18 (2.40)	55.8	09.1	50.7	40.2	110,186
Unemployed	5.60 (2.60)	03.6	09.7	61.5	28.8	7,010
Not active	5.55 (2.40)	40.6	06.8	63.0	30.2	80,166
Perception of income						
Very difficult	6.43 (2.60)	07.2	07.7	60.4	31.8	14,116
Difficult	5.98 (2.40)	20.8	08.5	59.6	31.9	41,044
Coping	5.32 (2.30)	45.6	08.3	58.0	33.7	89,948
Living comfortably	4.61 (2.20)	26.5	08.0	48.9	43.1	52,254
Total						197,362

Source: European Social Survey ((ESS 2018i; 2016; 2014; 2012; 2010; 2008; 2006; 2004; 2002)

The mean response reported above is derived from weighted data (dweight). Please see Appendix 1 for complete details about the variable construction. A replication package is available at [https://bit.ly/3ecrv4I]

### Covariates

The sex of the respondent as well as other sociodemographic characteristics (age, migrant status, urbanicity) are considered in the analysis. Most respondents are 45–64 with about a third (34.7%) younger and a quarter (23.7%) older. The sample is limited to those with citizenship, which include some migrants with about 6% and 7% being 1st or 2nd generation, respectively. Material conditions play a role in educational trajectories as well and employment status, delineating the employed (55.8%) from the unemployed (3.6%) and inactive (40.6%), and income perception, ranging from very difficult (7.2%) to living comfortably (26.5%), are taken into account. Table 2 also reports average perception of immigration. For example, those who report very difficult are notably more opposed to immigration relative to those living comfortably (6.43 vs. 4.61). Specific details about each measure are available via the associated replication package [https://bit.ly/3ecrv4I].

### The Diagonal Mobility Model: Overview and specification

Social mobility presents a number of methodological and conceptual challenges. The primary issue is identification. Mobility is dynamic in that it involves both origin status (e.g. parental education) and a destination status (i.e. one’s own education), which cannot be considered independently. The most common approach to date is the diagonal mobility model (DMM), sometimes referred to as the diagonal reference model (DRM), which emerged in the 1980s (Sobel, 1981, 1985). The innovation of the model is the possibility of separating the role of mobility from that of the origin and destination statuses and, as such, the DMM enjoys widespread usage in the literature on social, educational and occupational stratification (Van der Waal et al., 2017).

1 $$\mathrm{Yijk }=\mathrm{ pzii }+ (1 -\mathrm{ p})\mathrm{zjj }+\mathrm{ Mbxijb }+ \sum \mathrm{b \beta bxijb }+\mathrm{ \varepsilon ijk}$$

Equation 1 (E1) is the baseline model in which an outcome, attitudes towards immigration in this case, is determined by accounting for the relative weight/importance of parental education i (cell *ii* in the mobility table) and one’s own education j (cell *jj* in the mobility table). The relative importance of parental education is denoted by the weight *pzii* and, it follows, (1 − *p*)*j* identifies the relative importance of one’s own education, which are multiplied by the population means of the *ii*th and *jj*th cells in a mobility table. In all cases, the stochastic term, ε*ijk*, has an expectation of 0. E1 underlines the DMM’s ability to include covariates, ∑*b* β*bxijb*, and an independent measure of upward or downward mobility, *Mbxijb*, additively. Often, and with reason (Sobel, 1981, 1985), the inclusion the independent measure of mobility, *Mbxijb*, is interpreted as identifying the additive role of mobility in a given outcome of interest (for an example considering wellbeing see Schuck & Steiber, 2018).

Recently, evidence is emerging that the DMM does not necessarily offer an independent perspective on mobility and that it suffers from the same drawbacks as earlier approaches (e.g. Square Additive Model; Duncan, 1966) in that the origin, destination and mobility “effects” are constrained because of an implicit linearity assumed by the model (Fosse & Pfeffer, 2019). The result is that the model offers a limited test of significance for an independent role of mobility premised on a number of assumptions that implicitly constrain the estimate for mobility towards zero. This is a concern as, which has been pointed out by others (Fosse & Pfeffer, 2019), the DMM can leave the impression that mobility is of moderate or no consequence, but for reasons attributable to model specification.

Another drawback of considering independent tests of mobility is interpretative. The meaning of an independent “effect” in models of educational mobility is somewhat difficult to interpret as there are clear constraints on the extent to which mobility is possible. For example, depending on the educational context and measures available, children of tertiary education have no real upward mobility available to them. Similarly, children of primary-educated parents are only able to maintain or improve on their educational origins. In both cases, the implication of a significant association between upward mobility/downward mobility is of limited interpretative use as only some of the outcomes are plausible.

2 $$\mathrm{Yijk }=\mathrm{ pzii }+ (1 -\mathrm{ p})\mathrm{zjj }+ \sum \mathrm{b \beta bxijb }+\mathrm{ \varepsilon ijk}$$

In this work, the DMM model is used but the independent role of mobility is not considered independent from the estimates for parental and one’s own education. This reduced form of the DMM remains an insightful approach to ascertain the relative importance/weight of origin, *pzii*, and destination education, (1 − *p*)*j*, which is made explicit in Eq. 2 (E2). As these relative weights vary by context, the contribution of parental education and one’s own education to the outcome of interest—attitudes towards immigration—results in insightful and interpretable variation.

3 $$\mathrm{E}[\mathrm{Y}|\mathrm{X},\mathrm{ Z}] =\mathrm{ pZ }+ (1 -\mathrm{ p})\mathrm{Z }+ \sum \mathrm{b \beta bX}$$

Equation 3 (E3) underlines the ability of the parameters estimated by E2 to provide expected values for distinct combinations of origin and destination education so long as plausible values for any additional control variables are held constant in the model. Rather than deriving an independent estimate for educational mobility, comparing distinct mobility combinations (e.g. primary parental education to secondary individual education), defined by the pattern of expected values, offers unique insight into how educational mobility shapes perspectives on migrants. Distinct changes between contexts and intergenerationally shape meaningful variation within countries (e.g. primary-primary in Ireland vs. primary-tertiary in Ireland) and between countries (e.g. secondary-tertiary in Ireland vs. secondary-tertiary in Poland).

## Results

### Diagonal Mobility Model—All ESS Rounds/Years and Countries

Table 3 reports the estimated coefficients, associated origin/destination weights and sample/fit statistics for three nested diagonal mobility models (DMMs) assessing the association between attitudes towards immigration and educational mobility. The first model considers only the baseline association with educational mobility (origin and destination), net of the ESS round/year and country. The second model adds sociodemographic controls (age, sex, migrant status and urbanicity). Model 3, in addition to all measures included in models 1 and 2, adds economic controls (employment and perception of income). Of note, the magnitude of difference between the diagonals of the mobility table, termed the mobility effect, which assesses differences by type of stable intergenerational reproduction of educational attainment, attenuates only slightly with the inclusion of additional controls. In short, the inclusion of controls for perception of income and employment do not explain the pattern associated with mobility. In general, the estimated coefficients and tests of significance for mobility, as well as the associated weights, change little across models. Model 3, which is considered the full specification, is the preferred model for interpretation.

**Table 3:** Diagonal mobility model—all ESS rounds/years and countries

	Model 1 β(SD)	Model 2	Model 3
Diagonal effect (*Ref. Low-Low*)			
Stable Med.-Med	− 0.825 ^***^ (0.02)	− 0.751^***^ (0.02)	− 0.655^***^ (0.02)
Stable High-High	− 1.461 ^***^ (0.02)	− 1.355^***^ (0.02)	− 1.167^***^ (0.02)
Sex (*Ref. Female*)		− 0.051^***^ (0.01)	− 0.020^*^ (0.01)
Age		0.010*** (0.00)	0.005*** (0.00)
Migrant status (*Ref. Native*)			
1st generation		− 0.630^***^ (0.02)	− 0.693^***^ (0.02)
2nd generation		− 0.288^***^ (0.02)	− 0.310^***^ (0.02)
Urbanicity (*Ref. Rural*)		0.036*** (0.01)	0.035** (0.01)
Employment (*Ref. Employed*)			
Unemployed			0.042 (0.03)
Not active			0.050*** (0.01)
Perception of income (*Ref. very difficult*)			
Difficult			− 0.231^***^ (0.02)
Coping			− 0.497^***^ (0.02)
Living comfortably			− 0.818^***^ (0.02)
ESS Round/Year	Yes	Yes	Yes
Country Fixed Effects	Yes	Yes	Yes
Parent weight (origin)	0.24 (0.01)	0.21 (0.01)	0.21 (0.01)
Respondent weight (destination)	0.76 (0.01)	0.79 (0.01)	0.79 (0.01)
AIC	981,453	980,199	978,463
Observations	197,362	197,362	197,362

Source: European Social Survey ((ESS 2018i; 2016; 2014; 2012; 2010; 2008; 2006; 2004; 2002)

The estimates are derived from weighted data (dweight). Fixed effects are included for ESS round/year and country. A replication package is available at [https://bit.ly/3ecrv4I]

^*^p < 0.05, **p < 0.01, **p < 0.001

The primary insight of Table 3 is the consistent gradient across the diagonal of the mobility table. Each higher, stable mobility combination is associated with a significant and non-trivial reduction in the extent to which immigration is perceived negatively. For example, those with high education whose parents were similarly advantaged, report an average of 1.17 lower opposition to immigration, controlling for sociodemographic and economic attributes of the individual. Sex, age and migrant status are all significant with women, natives and older people significantly more likely to report greater opposition. The weights for the education of parents and respondent, which reflect the relative importance of parental (origin) or one’s own (destination) education in shaping the outcome of interest—attitudes towards immigration—are notably skewed towards destination states. Specifically, across all countries and years, 0.79 (also interpretable in percentage terms—79%) of the importance of education is determined by one’s own educational experience rather than that of their parents. What Table 3 cannot offer insight into is the variation across countries and the substantive meaning of the observed coefficients in terms of expected differences in anti-immigrant sentiment under distinct and plausible mobility trajectories.

### Diagonal Mobility Model—Expected Values by Country and Mobility Combination

**Fig. 1:**
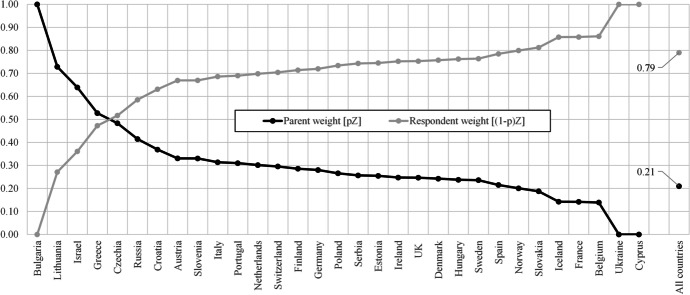
Weights for respondent and parent—all ESS rounds/years by country. Source: European Social Survey ((ESS 2018i; 2016; 2014; 2012; 2010; 2008; 2006; 2004; 2002). The weights, by country and overall, are derived from model 3 in Table 3. A replication package is available at [https://bit.ly/3ecrv4I]

Tables 4 and 5 use the estimated parameters in model 3 of Table 3 to estimate and compare the perception of immigration by all possible mobility combinations for each country included in the data. Figure 1 offers a summative visual to show the pattern of parental (origin) and respondent (destination) weights, sorted in descending order by the weight/influence of parental education. The expected values vary in non-trivial ways with a low of 2.38 (High-High; Iceland) to a high of 8.35 (Low-Low; Greece).^7^

**Table 4:** Expected values by mobility combination—all ESS rounds/years by country

Parent ed. (origin)	Expected values by mobility combination								
	Low	Med	High	Low	Med	High	Low	Med	High
Resp. ed. (destination)	Low	Low	Low	Med	Med	Med	High	High	High
	E[Y|X,Z]								
Bulgaria	3.94	4.13	4.06	3.94	4.13	4.06	4.13	3.94	4.06
Lithuania	5.42	5.31	5.00	5.38	5.26	4.96	5.15	5.26	4.85
Israel	7.57	7.20	6.91	7.36	6.99	6.70	6.83	7.20	6.54
Greece	8.35	7.93	7.68	7.98	7.56	7.31	7.33	7.75	7.08
Czechia	6.03	5.88	5.82	5.87	5.72	5.66	5.65	5.80	5.58
Russia	7.08	7.03	6.92	7.01	6.96	6.85	6.81	6.86	6.70
Croatia	5.84	5.67	5.48	5.54	5.37	5.19	5.06	5.23	4.87
Austria	7.13	6.93	6.65	6.73	6.53	6.25	5.95	6.15	5.66
Slovenia	6.89	6.61	6.29	6.33	6.06	5.73	5.41	5.68	5.08
Italy	6.74	6.47	6.24	6.15	5.89	5.65	5.38	5.64	5.14
Portugal	6.44	6.30	6.10	6.13	5.99	5.79	5.54	5.68	5.34
Netherlands	5.79	5.65	5.57	5.47	5.33	5.24	5.13	5.27	5.05
Switzerland	5.63	5.46	5.30	5.23	5.06	4.89	4.67	4.84	4.51
Finland	6.07	5.93	5.68	5.72	5.58	5.33	4.94	5.08	4.68
Germany	5.94	5.70	5.54	5.34	5.10	4.94	4.68	4.92	4.52
Poland	4.65	4.58	4.51	4.46	4.39	4.32	4.20	4.27	4.13
Serbia	5.46	5.49	5.25	5.53	5.55	5.31	4.86	4.84	4.62
Estonia	5.58	5.48	5.37	5.30	5.20	5.09	4.87	4.97	4.76
Ireland	6.83	6.64	6.41	6.26	6.07	5.84	5.37	5.56	5.14
UK	6.67	6.48	6.13	6.09	5.90	5.55	4.84	5.03	4.50
Denmark	4.62	4.39	4.20	3.91	3.69	3.49	3.09	3.31	2.89
Hungary	6.90	6.78	6.65	6.52	6.40	6.27	5.98	6.10	5.85
Sweden	4.95	4.77	4.60	4.38	4.21	4.04	3.66	3.83	3.49
Spain	5.65	5.54	5.40	5.26	5.16	5.02	4.66	4.76	4.52
Norway	6.01	5.96	5.80	5.82	5.77	5.60	5.11	5.15	4.94
Slovakia	5.80	5.76	5.74	5.63	5.59	5.57	5.51	5.55	5.49
Iceland	3.09	3.04	2.99	2.82	2.77	2.72	2.44	2.48	2.38
France	5.30	5.17	5.10	4.53	4.41	4.34	3.97	4.10	3.90
Belgium	6.50	6.47	6.37	6.26	6.23	6.13	5.61	5.65	5.51
Ukraine	5.93	5.93	5.93	5.61	5.61	5.61	5.32	5.32	5.32
Cyprus	6.92	6.92	6.92	6.95	6.95	6.95	6.32	6.32	6.32

Source: European Social Survey ((ESS 2018i; 2016; 2014; 2012; 2010; 2008; 2006; 2004; 2002)

The reported expected values are derived from model 3 in Table 5. For covariates other than education, the mean observed response or proportion, in the case of dichotomous measures, is used. A replication package is available at [https://bit.ly/3ecrv4I]

**Table 5:** Expected difference by mobility combination and weights for respondent and parent—all ESS rounds/years by country

Expected difference between select combinations				Weights			
	Low–High vs. Med-High	Low–High vs. High-High	Med-High vs. High-High	Parent (origin) pZ (SD)	Respondent (destination) (1-p)Z (SD)		Observations
Bulgaria	0.19	0.07	− 0.13	1.00 (0.00)	0.00	(0.00)	5039
Lithuania	− 0.11	0.30	0.41	0.73 (0.12)	0.27	(0.12)	5503
Israel	− 0.37	0.29	0.66	0.64 (0.07)	0.36	(0.07)	6290
Greece	− 0.41	0.26	0.67	0.53 (0.10)	0.47	(0.10)	2095
Czechia	− 0.15	0.07	0.22	0.48 (0.12)	0.52	(0.12)	9834
Russia	− 0.05	0.11	0.16	0.41 (0.19)	0.59	(0.19)	6887
Croatia	− 0.17	0.19	0.36	0.37 (0.17)	0.63	(0.17)	2237
Austria	− 0.20	0.29	0.49	0.33 (0.07)	0.67	(0.07)	4954
Slovenia	− 0.27	0.32	0.60	0.33 (0.05)	0.67	(0.05)	6035
Italy	− 0.27	0.23	0.50	0.31 (0.07)	0.69	(0.07)	4655
Portugal	− 0.14	0.20	0.34	0.31 (0.09)	0.69	(0.09)	7194
Netherlands	− 0.14	0.08	0.22	0.30 (0.06)	0.70	(0.06)	8378
Switzerland	− 0.17	0.16	0.33	0.30 (0.06)	0.70	(0.06)	6190
Finland	− 0.14	0.26	0.40	0.29 (0.04)	0.71	(0.04)	8177
Germany	− 0.23	0.16	0.40	0.28 (0.03)	0.72	(0.03)	12,853
Poland	− 0.07	0.07	0.14	0.27 (0.15)	0.73	(0.15)	7097
Serbia	0.02	0.24	0.22	0.26 (0.30)	0.74	(0.30)	1593
Estonia	− 0.10	0.11	0.21	0.25 (0.08)	0.75	(0.08)	6918
Ireland	− 0.19	0.23	0.42	0.25 (0.04)	0.75	(0.04)	8798
UK	− 0.19	0.35	0.54	0.25 (0.03)	0.75	(0.03)	7581
Denmark	− 0.23	0.19	0.42	0.24 (0.04)	0.76	(0.04)	4846
Hungary	− 0.12	0.13	0.25	0.24 (0.09)	0.76	(0.09)	6321
Sweden	− 0.17	0.17	0.34	0.24 (0.04)	0.76	(0.04)	5119
Spain	− 0.11	0.14	0.24	0.22 (0.07)	0.78	(0.07)	7646
Norway	− 0.05	0.17	0.22	0.20 (0.05)	0.80	(0.05)	6970
Slovakia	− 0.04	0.02	0.06	0.19 (0.27)	0.81	(0.27)	4239
Iceland	− 0.04	0.06	0.10	0.14 (0.19)	0.86	(0.19)	1250
France	− 0.13	0.07	0.20	0.14 (0.05)	0.86	(0.05)	8,784
Belgium	− 0.04	0.10	0.14	0.14 (0.06)	0.86	(0.06)	7,640
Ukraine	0.00	0.00	0.00	0.00 (0.00)	1.00	(0.00)	3,978
Cyprus	0.00	0.00	0.00	0.00 (0.00)	1.00	(0.00)	2,343
Total				0.21 (0.01)	0.79	(0.01)	197,362

Source: European Social Survey ((ESS 2018i; 2016; 2014; 2012; 2010; 2008; 2006; 2004; 2002)

The differences are calculated using the reported expected values in Table 6, which are derived from model 3 in Table 5. Reported weights, by country and overall, are derived from model 3 in Table 5. A replication package is available at [https://bit.ly/3ecrv4I]

As mentioned, when the model was introduced, we do not estimate parameters or expected values for mobility as an independent construct. Instead, using E2 and E3, we evaluate the meaning of mobility by comparing plausible mobility trajectories overall and by country. These patterns are determined by two main factors. First, the estimated parameters for the diagonal in the mobility table (see Table 3). This varies by country depending on the observed perception of immigration and the patterning of education by origin and destination (see E2). Second, the estimated weight/influence of parental and respondent determines the extent to which attitudes towards immigration shift depending on the origin and destination mobility combination.

Table 5 offers a convenient summary of the expected change in the perception of immigration by educational mobility type, focusing on those that end up with tertiary education (i.e. high). One insightful case is that of Italy (Table 5, column 3), where those the exceed their parents’ education by two levels (i.e. Low–High) are expected to have 0.27 less opposition to immigration relative to those who only exceeded their parents by one level (i.e. Med.-High). This is illustrative, but not unique. Nearly all countries experience a similar pattern in that one’s own education, if resulting from high levels of mobility, moderates attitudes towards immigration more than those whose parents’ where medium in terms of education.^8^

When contrasting different mobility types to stability among the highly educated (e.g. Med.-High vs. High-High, Low–High vs. High-High) the difference is almost uniformly positive. The implication is that for any origin education below high (i.e. Med., Low), no type of mobility will result in an expected perception of immigration more positive than that originate from parents with high levels of schooling. In other words, mobility is distinctly meaningful for understanding differences between those that experience some mobility rather than among those that are stable, particularly among the most educated.

## Discussion

Let us consider the implications of these findings for the hypotheses driving this work. Do the observed patterns offer insight into the role of mobility in shaping attitudes towards migrants? In regard to the first hypothesis,^9^ the short answer is yes—although not without caveats. The educational origin category that offers plausible upward and downward mobility falls in the middle (i.e. upper secondary), which is a reasonable basis from which to draw conclusions. For most countries, opposition to immigration falls in the presence of upward mobility. However, there are notable exceptions in contexts where parental education is more influential—Bulgaria (1.00), Lithuania (0.73), Israel (0.64), Greece (0.53) and the Czech Republic (0.48). In other words, we conclude that mobility can result in a less restrictive posture towards immigration, but only when the weight of one’s own schooling is relatively more influential.

The meaning of parental education carrying greater weight/influence speaks to a second set of hypotheses.^10^ When considering variation in the importance of mobility, countries where parental education matters, on average, experience less change in attitudes towards immigration. The contexts most heavily weighted towards parents—again, Bulgaria, Lithuania, Israel, Greece and the Czech Republic—experience an average increase in opposition to immigration of 0.16 for those downwardly mobile (i.e. medium to low) relative to those reproducing their parents’ level of schooling (i.e. medium to medium). For these same countries, the upwardly mobile are expected to also experience a decline in their reported opposition to immigration of 0.06, which is close to zero—indicating that mobility is not a large determinant of the perception of immigration.

Compare this pattern to the remaining contexts where one’s own education, relative to their parents’, matters more. In these contexts, the expected average increase in opposition to immigration is 0.36 with countries like France, Germany and Sweden expected to experience larger changes of 0.77, 0.60 and 0.56 respectively. This pattern, in line with the expectation (H2b) that a greater gap in sentiment should be present in contexts where one’s own education matters more, suggests that mobility is more substantively important where educational destinations carry greater weight. In general, we conclude that mobility does matter and variation in mobility can help us understand the role of education in shaping attitudes towards immigration. However, it is important avoid over-generalising.

In some countries where own’s own education is, relative to one’s parent, more influential, we observe only modest reductions in negative perceptions of immigration. Poland, for example, declines by 0.12 in terms of expected attitudes towards immigration and the Netherlands only 0.06. We contend that, overall, mobility moderates opposition to immigration. However, we suggest that country-context can, in some cases, matter more. This has implications for policy and future work.

## Implications

We suggest two ways forward with this line of research. First, we suggest that work should consider intergenerational normative change. Although this work is a reasonable first step to understand intergenerational socialisation dynamics and their role in shaping attitudes towards immigration, it focuses on education without unpacking the more proximate mechanisms that might be at play. Contexts with large and/or rapid changes in norms, defined by notable generation gaps in a variety of behaviours (e.g. voting preferences, occupational trajectories, family dynamic and a host of attitudinal frames) would theoretically be where destination education, if influential, could be of notably greater importance. Second, this work should focus on cohorts that reflect both policy and economic periods of expansion and contraction. To offer a general, first perspective, we have looked at expected mobility patterns net of cohort and period. However, it is meaningful to explore change over time.

Aside from providing a clear pathway forward in the academic literature, the results of this work have clear implications for policy. This work shows variation in the extent to which policy that improves educational access could be expected to significantly impact patterns of anti-immigrant sentiment. In contexts where parental education carries a greater weight (e.g. Lithuania, Greece), targeting interventions that facilitate educational attainment for current or expectant parents would be expected to translate into attitudinal change for the next generation. In contexts where parental influence is weaker, although improving educational conditions for all is a reasonable goal unto itself, targeting children’s educational trajectory would be a more plausible pathway towards reducing anti-immigrant sentiment.

Of note, evidence suggests that there is meaningful variation in the attitudes and the role of educational mobility across regions in Europe. Specifically, as the discussion highlighted, France, Germany and Sweden are countries where individual mobility matters more and are expected to experience larger changes in attitudes across levels of mobility. Of note, these countries are also located in the more Western part of Europe. Eastern and Southern Europe (e.g. Bulgaria, Lithuania, Israel, Greece and the Czech Republic) exhibit a different pattern—more weighted towards parental education with overall greater levels of antipathy. While this pattern does not always hold, future work should be sensitive to these regional cleavages and work to better capture their economic and policy contexts.

That said, this work is only a first step—albeit is necessary—as existing policy conditions are only indirectly considered as a function of age, which is only minimally descriptive. Specifically, the need for a clearer accounting of the policy environment, which takes into account differences in terms of impact for parents and children, is the best way to understand what mobility means for shaping (in)tolerance in contexts of reception. This includes analysis that better captures the pathways by which previous generations, both parents and grandparents, contribute to child socialisation into values via physical proximity or other forms of influence. These two suggestions (i.e. policy and influence) follow suggestions in the stratification literature focused on educational mobility more generally (Torche, 2015).

In sum, this work offers insight into the patterning of educational mobility and anti-immigrant sentiment in a variety of European contexts and Israel. If a single detail has emerged that deserves to be underlined, it is that the role of education in moderating antipathy towards migrants is defined by multiple generations. This is an important addition to recent work that suggests education can moderate a reaction of intolerance in moments of rapid demographic change like a historically large influx of refugees (Velásquez & Eger, 2022). Of note, these data preceded the COVID-19 pandemic, which also has implications in terms of patterning movement and economic conditions. In the end, it is clearly not just about upward or downward inter-generational mobility, but is better understood as a process of socialisation that is differently shaped by the relative importance of parental and one’s own experience. We see this as an important contribution and a step beyond models of anti-immigrant sentiment rooted entirely in material and socio-cultural threat. This does not suggest that educational mobility is the only determinant of meaning nor, in some cases, the most influential. However, it does point to an understanding of attitudinal formation that is more closely linked to the life course.

## Appendix 1

**Table 6:** Valid and missing cases by variable

Variable	Valid (Initial sample)	Missing (*n*)	Missing (%)
Opposition to immigration	222,583	10,633	5%
Respondents’ educational level (eisced)	232,731	485	0%
Parent's educational level (eiscedm or eiscedf)	209,418	23,798	10%
Age	233,216	–	0%
Sex	233,148	68	0%
Migrant status	233,156	60	0%
Urbanicity	232,824	392	0%
Employment	232,485	731	0%
Perception of income	230,947	2269	1%

Source: European Social Survey ((ESS 2018i; 2016; 2014; 2012; 2010; 2008; 2006; 2004; 2002)

Table 6 reports the number of valid missing (*n* and %) by variables (see Table 2 for descriptive statistics). The initial sample is of all respondents to rounds 4–9 of the European Social Survey ((ESS 2018i; 2016; 2014; 2012; 2010; 2008; 2006; 2004; 2002) who are aged 26 years or older and citizens of the country in which the survey was conducted. Three variables constitute the majority of the missing cases—opposition to immigration (10,633), parental education (23,798) and perception of income (2269). A respondent is excluded if any measure is missing, which results in a final analytic sample of 197,362.

**Table 7:** Cases selection from original sample to analytic sample

Case selection	Sample
Original ESS cumulative dataset (1–9)	**417,533**
↳ Rounds 4 to 9	287,701
↳ Aged 26 +	242,195
↳ Citizens (Initial sample)	**233,216**
↳ Valid outcome variable	222,583
↳Valid predictor	200,294
↳Valid covariates (Final analytic sample)	**197,362**

Source: European Social Survey ((ESS 2018i; 2016; 2014; 2012; 2010; 2008; 2006; 2004; 2002)

The bold numbers are for (1) the initial full dataset, (2) the initial sample after round and age selection and (3) the final analytic sample

Table 7 reports the case selection from the initial cumulative dataset (i.e. all rounds of the European Social Survey), which consists of 9 rounds, to the final analytic sample used to estimate the diagonal mobility models. The selection process is reported sequentially. The first selection involves limiting the analysis to rounds 4 to 9, which are the rounds in which respondent and parental education are collected and comparable across countries and time. Age selection follows, which excludes respondents younger than 26 to prevent conflating complete with ongoing education. Finally, respondents are limited to citizens of the country where the data was collected. The resulting initial sample (233,216) includes all respondents to rounds 4–9 of the European Social Survey (ESS 2018i; 2016; 2014; 2012; 2010; 2008; 2006; 2004; 2002) who are aged 26 years or older and citizens of the country in which the survey was conducted. The final stage consists of a listwise deletion of observations with missing data for the predictor variable and covariates.

### Replication package

A complete replication package is available (https://bit.ly/3ecrv4I). The coding for each measure used and the steps outlined in Table 6 and 7 can be reproduced.

## Footnotes

1 Recent evidence suggests that the DMM/DRM suffers from strong assumption about linearity in independent effect of mobility (Fosse & Pfeffer, 2019), which we consider in this work.

2 Although not specifically considering tolerance, there is evidence that intergenerational mobility can shape more broadly such as child-rearing (Sieben 2017).

3 New users can register at the following URL: https://www.europansocialsurvey.org/user/new

4 “The production of ESICED is particularly dependent on the availability of sufficiently detailed country specific education variables. Hence, for ESS1-3 EISCED has only been produced for respondents, but not for all countries. In ESS4 the availability of country specific education variables has improved and EISCED has been produced also for partner, father and mother (ESS 2009, p. 5).”.

5 ISCED I (less than lower secondary; low), ISCED II (lower secondary; low), ISCED IIIb (lower-tier upper secondary; medium), ISCED IIIa (upper-tier upper secondary; medium), ISCED IV advanced vocational; medium), ISCED V1 (lower tertiary; high) and ISCED V2 (higher tertiary; high).

6 (Q1) Would you say it is generally bad or good for [country]’s economy that people come to live here from other countries?; (Q2) Would you say that [country]’s cultural life is generally undermined or enriched by people coming to live here from other countries?; (Q3) Is [country] made a worse or a better place to live by people coming to live here from other countries?

7 The estimates for Ukraine and Cyprus do not change by parental education and, therefore, respondents’ education wholly determine the outcome—reflected in a respondent/destination weight of 1 (see Fig. 1 and the last two rows of Table 7).

8 The exceptions are Bulgaria, which has the highest influence of parental education with a parental weight of 1 (compared to 0.21 overall), Serbia, which has a notably flat gradient, and the two countries (Ukraine and Cyprus) with no change as parental weights are zero.

9 Individuals who have attained the same or less education than their parents are expected to be less supportive of immigration that those who have experienced upward educational mobility (H1).

10 Educational mobility matters less, reflected in a smaller gap between those who do and do not experience upward mobility, in moderating attitudes toward migrants in contexts where parental education is relatively more influential (H2a). Educational mobility matters more, reflected in a greater gap between those who do and do not experience upward mobility, in moderating attitudes toward migrants in contexts where parental education is relatively less influential (H2b).

## References

[CR1] Alwin DF, Krosnick JA (1991). Aging, cohorts, and the stability of sociopolitical orientations over the life span. American Journal of Sociology.

[CR2] Arel-Bundock V, Pelc K (2018). When can multiple imputation improve regression estimates?. Political Analysis.

[CR3] Bazo-Vienrich A, Creighton MJ (2017). What’s left unsaid? In-group solidarity and ethnic and racial differences in opposition to immigration in the United States. Journal of Ethnic and Migration Studies.

[CR4] Becker GS, Kominers SD, Murphy KM, Spenkuch JL (2018). A Theory of Intergenerational Mobility. Journal of the Political Economy.

[CR5] Beller E (2009). Bringing intergenerational social mobility research into the twenty-first century: Why mothers matter. American Sociological Review.

[CR6] Bentsen BMA (2022). Intergroup contact and negative attitudes towards immigrants among youth in Sweden: Individual and contextual factors. International Migration & Integration.

[CR7] Björklund, A., & Salvanes, K. G. (2011). Education and family background: Mechanisms and policies. In E.A. Hanushek, S. Machin, & L. Woessmann (Eds.), *Handbook of the Economics of the Education* (3, 201–247). Elsevier.

[CR8] Blalock HM (1967). Status inconsistency, social mobility, status integration and structural effects. American Sociological Review.

[CR9] Blau PM (1956). Social mobility and interpersonal relations. American Sociological Review.

[CR10] Blau PM, Duncan OD (1967). The American occupational structure.

[CR11] Bobo L, Licari FC (1989). Education and political tolerance: Testing the effects of cognitive sophistication and target group affect. Public Opinion Quarterly.

[CR12] Borgonovi F, Pokropek A (2019). Education and attitudes toward migration in a cross country perspective. Frontiers in Psychology.

[CR13] Borjas, G. J. (1999). The economic analysis of immigration. In D. Card & O. Ashenfelter (Eds.), *Handbook of Labour Economics* (3 1697–1760). Elsevier Science B.V.

[CR14] Bourdieu P, Richardson J (1986). The forms of capital. Handbook of Theory and Research for the Sociology of Education.

[CR15] Breen R (2001). Social mobility and constitutional and political preferences in Northern Ireland. The British Journal of Sociology.

[CR16] Capistrano D (2020). Education and support for a culture of peace: A critical comparative analysis using survey data. Global Change, Peace & Security.

[CR17] Ceobanu AM, Escandell X (2010). Comparative analyses of public attitudes toward immigrants and immigration using multinational survey data: A review of theories and research. Annual Review of Sociology.

[CR18] Chan TW (2018). Social mobility and the well-being of individuals: Social mobility and well- being. The British Journal of Sociology.

[CR19] Cordero-Coma J, Esping-Andersen G (2018). Parental time dedication and children’s education. An analysis of West Germany. Research in Social Stratification and Mobility.

[CR20] Creighton MJ, Jamal A (2021). An overstated welcome: Brexit and intentionally masked anti-immigrant sentiment in the UK. Journal of Ethnic and Migration Studies.

[CR21] Creighton MJ, Jamal A, Malancu NC (2015). Has opposition to immigration increased in the United States after the economic crisis?. An Experimental Approach, International Migration Review.

[CR22] Creighton MJ, Schmidt P, Zavala-Rojas D (2019). Race, wealth and the masking of opposition to immigrants in the Netherlands. International Migration.

[CR23] Creighton MJ, Fahey É, McGinnity F (2022). Immigration, identity and anonymity: Intentionally masked intolerance in Ireland. International Migration Review.

[CR24] Dalhouse M, Frideres JS (1996). Intergenerational congruency: The role of the family in political attitudes of youth. Journal of Family Issues.

[CR25] Darling N, Steinberg L (1993). Parenting style as context: An integrative model. Psychological Bulletin.

[CR26] Duncan OD, Smelser NL, Lipset SM (1966). Methodological issues in the analysis of social mobility. Social Structure and Mobility in Economic Development.

[CR27] Erola J, Jalonen S, Lehti H (2016). Parental education, class and income over early life course and children's achievement. Research in Social Stratification and Mobility.

[CR28] ESS Round 8: European Social Survey (2018a): ESS-8 2016 documentation report. Edition 2.1. Bergen, European Social Survey Data Archive, NSD – Norwegian Centre for Research Data for ESS ERIC. 10.21338/NSD-ESS9-2016.

[CR29] ESS Round 7: European Social Survey (2018b): ESS-7 2014 documentation report. Edition 3.2. Bergen, European Social Survey Data Archive, NSD – Norwegian Centre for Research Data for ESS ERIC. 10.21338/NSD-ESS9-2014.

[CR30] ESS Round 6: European Social Survey (2018c): ESS-6 2012 documentation report. Edition 2.4. Bergen, European Social Survey Data Archive, NSD – Norwegian Centre for Research Data for ESS ERIC. 10.21338/NSD-ESS9-2012.

[CR31] ESS Round 5: European Social Survey (2018d): ESS-5 2010 documentation report. Edition 4.2. Bergen, European Social Survey Data Archive, NSD – Norwegian Centre for Research Data for ESS ERIC. 10.21338/NSD-ESS9-2010.

[CR32] ESS Round 4: European Social Survey (2018e): ESS-4 2008 Documentation Report. Edition 5.5. Bergen, European Social Survey Data Archive, NSD – Norwegian Centre for Research Data for ESS ERIC. 10.21338/NSD-ESS9-2008.

[CR33] ESS Round 9: European Social Survey (2019): ESS-9 2018 documentation report. Edition 1.2. Bergen, European Social Survey Data Archive, NSD – Norwegian Centre for Research Data for ESS ERIC. 10.21338/NSD-ESS9-2018.

[CR34] ESS Round 9: European Social Survey Round 9 Data (2018i). Data file edition 1.1. NSD – Norwegian Centre for Research Data, Norway – Data archive and distributor of ESS data for ESS ERIC. 10.21338/NSD-ESS9-2018i.

[CR35] ESS Round 4: European Social Survey Round 4 Data (2008). Data file edition 4.5. NSD – Norwegian Centre for Research Data, Norway – Data archive and distributor of ESS data for ESS ERIC. 10.21338/NSD-ESS4-2008.

[CR36] ESS Round 6: European Social Survey Round 6 Data (2012). Data file edition 2.4. NSD – Norwegian Centre for Research Data, Norway – Data archive and distributor of ESS data for ESS ERIC. 10.21338/NSD-ESS6-2012.

[CR37] ESS Round 7: European Social Survey Round 7 Data (2014). Data file edition 2.2. NSD – Norwegian Centre for Research Data, Norway – Data archive and distributor of ESS data for ESS ERIC. 10.21338/NSD-ESS7-2014.

[CR38] ESS Round 8: European Social Survey Round 8 Data (2016). Data file edition 2.1. NSD – Norwegian Centre for Research Data, Norway – Data archive and distributor of ESS data for ESS ERIC. 10.21338/NSD-ESS8-2016.

[CR39] ESS (2009). Education upgrade ESS1-ESS4 documentation report. Bergen: Norwegian Social Science Data Services.

[CR40] Finseraas H, Skorge O, Strøm M (2018). Does education affect immigration attitudes? Evidence from an education reform. Electoral Studies.

[CR41] Fosse, E., & Pfeffer, F. T. (2019). Beyond the diagonal reference model: Critiques and new directions. Presentation to the Population Association of America: Austin, Texas (12/4/2019). http://paa2019.populationassociation.org/abstracts/190797

[CR42] Friedman S (2016). Habitus clivé and the emotional imprint of social mobility. The Sociological Review (keele).

[CR43] Goldthorpe, J. H., Llewellyn, C., & Payne, C. (1987). Social mobility and class structure in modern Britain (2nd ed.) Claredon Press.

[CR44] Gorodzeisky A, Semyonov M (2020). Perceptions and misperceptions: Actual size, perceived size and opposition to immigration in European societies. Journal of Ethnic and Migration Studies.

[CR45] Hainmueller J, Hiscox MJ (2007). Educated preferences: Explaining attitudes toward immigration in Europe. International Organization.

[CR46] Hainmueller J, Hiscox MJ (2010). Attitudes toward highly skilled and low-skilled immigration: Evidence from a survey experiment. American Political Science Review.

[CR47] Hainmueller J, Hopkins DJ (2014). Public attitudes toward immigration. Annual Review of Political Science.

[CR48] Hauser RM, Featherman DL (1976). Equality of schooling: Trends and prospects. Sociology of Education.

[CR49] Hello E, Scheepers P, Gijsberts M (2002). Education and ethnic prejudice in Europe: Explanations for cross-national variances in the educational effect on ethnic prejudice. Scandinavian Journal of Educational Research.

[CR50] Hello E, Scheepers P, Vermulst A, Gerris JRM (2004). Association between educational attainment and ethnic distance in young adults: Socialization by schools or parents?. Acta Sociologica.

[CR51] Hello E, Scheepers P, Sleegers P (2006). Why the more educated are less inclined to keep ethnic distance: An empirical test of four explanations. Ethnic and Racial Studies.

[CR52] Hendrickx J, de Graaf ND, Lammers J, Ultee W (1993). Models for status inconsistency and mobility: A comparison of the approaches by Hope and Sobel with the mainstream square additive model. Quality and Quantity.

[CR53] Hess, R., & Torney, J. (1967). The development of political attitudes in children. Aldine Publishing Company

[CR54] Hope K (1971). Social mobility and fertility. American Sociological Review.

[CR55] Houle JN, Martin MA (2011). Does intergenerational mobility shape psychological distress? Sorokin revisited. Research in Social Stratification and Mobility.

[CR56] Jackson M, Grusky DB (2018). A post-liberal theory of stratification. The British Journal of Sociology.

[CR57] Jenssen AT, Engesbak H (1994). The many faces of education: Why are people with lower education more hostile towards immigrants than people with higher education?. Scandinavian Journal of Education Research.

[CR58] Knudsen K (1995). The education–tolerance relationship: Is it biased by social desirability?. Scandinavian Journal of Educational Research.

[CR59] Kratz K (2021). Do concerns about immigration change after adolescence?. How Education and Critical Life Events Affect Concerns about Immigration, European Sociological Review.

[CR60] Kunovich RM (2004). Social structural position and prejudice: An exploration of cross-national differences in regression slopes. Social Science Research.

[CR61] Kuppens T, Spears R (2014). You don’t have to be well-educated to be an aversive racist, but it helps. Social Science Research.

[CR62] Malloy B, Ozkok Z, Rosborough J (2021). The impact of immigration attitudes on voting preferences: Evidence from the European Social Survey. International Migration & Integration.

[CR63] Manevska K, Achterberg P (2013). Immigration and perceived ethnic threat: Cultural capital and economic explanations. European Sociological Review.

[CR64] Margaryan S., Paul A., Siedler T. (2019). Does education affect attitudes towards immigration? Evidence from Germany. *Journal of Human Resources*, 0318–9372R1.

[CR65] Mayda AM (2006). Who is against immigration? A cross-country investigation of individual attitudes towards immigrants. Review of Economic and Statistics.

[CR66] McLaren LM (2003). Anti-immigrant prejudice in Europe: Contact, threat perception, and preferences for the exclusion of migrants. Social Forces.

[CR67] McLaren LM, Johnson M (2007). Resources, group conflict and symbols: Explaining anti- immigration hostility in Britain. Political Studies.

[CR68] Miklikowska M (2017). Development of anti-immigrant attitudes in adolescence: The role of parents, peers, intergroup friendships, and empathy. British Journal of Psychology.

[CR69] Miller RB, Glass J (1989). Parent-child attitude similarity across the life course. Journal of Marriage and the Family.

[CR70] Min J, Silverstein M, Lendon JP (2012). Intergenerational transmission of values over the family life course. Advances in Life Course Research.

[CR71] Moen P, Erickson MA, Dempster-McClain D (1997). Their mother’s daughters? The intergenerational transmission of gender attitudes in a world of changing roles. Journal of Marriage and Family.

[CR72] Nikolaev B, Burns A (2014). Intergenerational mobility and subjective well-being: Evidence from the general social survey. Journal of Behavioural and Experimental Economics.

[CR73] Paskov M, Präg P, Richards L (2021). Does downward social mobility make people more hostile towards immigrants?. Research in Social Stratification and Mobility.

[CR74] Paulson EL (2018). A habitus divided? The effects of social mobility on the habitus and consumption. The European Journal of Marketing.

[CR75] ESS Round 5: European Social Survey Round 5 Data (2010). Data file edition 3.4. NSD – Norwegian Centre for Research Data, Norway – Data archive and distributor of ESS data for ESS ERIC. 10.21338/NSD-ESS5-2010.

[CR76] Rustenbach E (2010). Sources of negative attitudes toward immigrants in Europe: A multi-level analysis. International Migration Review.

[CR77] Scherger S, Savage M (2010). Cultural transmission, educational attainment and social mobility. The Sociological Review (keele).

[CR78] Scheve K, Slaughter M (2001). Labor market competition and individual preferences over immigration policy. Review of Economics and Statistics.

[CR79] Schröder H, Ganzeboom HBG (2014). Measuring and modelling level of education in European societies. European Sociological Review.

[CR80] Schuck B, Steiber N (2018). Does intergenerational educational mobility shape the well- being of young Europeans? Evidence from the European Social Survey. Social Indicators Research.

[CR81] Sieben I (2017). Child-rearing values: The impact of intergenerational class mobility. International Socioly.

[CR82] Sieben I, Huinink J, de Graaf PM (2001). Family background and sibling resemblance in educational attainment, trends in the former FRG, the former GDR, and the Netherlands. European Sociological Review.

[CR83] Sniderman PM, Hagendoorn L, Prior M (2004). Predispositional factors and situational triggers: Exclusionary reactions to immigrant minorities. American Political Science Review.

[CR84] Sniderman P. M., & Hagendoorn, L. (2007). When ways of life collide: Multiculturalism and its discontents in the Netherlands. Princeton University Press

[CR85] Sobel ME (1981). Diagonal mobility models: A substantively motivated class of designs for the analysis of mobility effects. American Sociological Review.

[CR86] Sobel ME (1985). Social mobility and fertility revisited: Some new models for the analysis of the mobility effects hypothesis. American Sociological Review.

[CR87] Sorokin, P. A. (1927). Social mobility. Harper & Brothers.

[CR88] Stawarz, N., & Müller, M. (2020). Concerns regarding immigration in Germany: how subjective fears, becoming unemployed and social mobility change anti-immigrant attitudes. *European Societies*,* 22*(3), 293–316.

[CR89] Strabac Z, Listhaug O (2008). Anti-Muslim prejudice in Europe: A multilevel analysis of survey data from 30 countries. Social Science Research.

[CR90] Tolsma K, de Graaf ND, Quillian L (2009). Does intergenerational social mobility affect antagonistic attitudes towards ethnic minorities?. The British Journal of Sociology.

[CR91] Torche F (2015). Intergenerational mobility and equality of opportunity. European Journal of Sociology.

[CR92] Van der Slik FWP, de Graaf ND, Gerris JRM (2002). Conformity to parental rules: Asymmetric influences of father’s and mother’s levels of education. European Sociological Review.

[CR93] Van der Waal J, Daenekindt S, de Koster W (2017). Statistical challenges in modelling the health consequences of social mobility: The need for diagonal reference models. International Journal of Public Health.

[CR94] Velásquez P, Eger MA (2022). Does higher education have liberalizing or inoculating effects? A panel study of anti-immigrant sentiment before, during, and after the European migration crisis. European Sociological Review.

[CR95] Weakliem DL (1992). Does social mobility affect political behaviour?. European Sociological Review.

[CR96] Weil FD (1985). The variable effects of education on liberal attitudes: A comparative-historical analysis of anti-Semitism using public opinion survey data. American Sociological Review.

[CR97] Zang E, de Graaf ND (2016). Frustrated achievers or satisfied losers? Inter- and intragenerational social mobility and happiness in China. Sociological Science.

[CR98] Zeng Z, Xie Yu (2014). The effects of grandparents on children’s schooling: Evidence from rural China. Demography.

